# Stochastically driven adult–recruit associations of tree species on Barro Colorado Island

**DOI:** 10.1098/rspb.2014.0922

**Published:** 2014-09-07

**Authors:** Stephan Getzin, Thorsten Wiegand, Stephen P. Hubbell

**Affiliations:** 1 Department of Ecological Modelling, Helmholtz Centre for Environmental Research—UFZ, Permoserstrasse 15, Leipzig 04318, Germany; 2 Department of Ecology and Evolutionary Biology, University of California, Los Angeles, CA 90095, USA; 3 Smithsonian Tropical Research Institute, Apartado 0843-03092, Balboa, Ancon, Republic of Panama

**Keywords:** Berman test, habitat association, life trait, pattern reconstruction, point pattern analysis, segregation

## Abstract

The spatial placement of recruits around adult conspecifics represents the accumulated outcome of several pattern-forming processes and mechanisms such as primary and secondary seed dispersal, habitat associations or Janzen–Connell effects. Studying the adult–recruit relationship should therefore allow the derivation of specific hypotheses on the processes shaping population and community dynamics. We analysed adult–recruit associations for 65 tree species taken from six censuses of the 50 ha neotropical forest plot on Barro Colorado Island (BCI), Panama. We used point pattern analysis to test, at a range of neighbourhood scales, for spatial independence between recruits and adults, to assess the strength and type of departure from independence, and its relationship with species properties. Positive associations expected to prevail due to dispersal limitation occurred only in 16% of all cases; instead a majority of species showed spatial independence (≈73%). Independence described the placement of recruits around conspecific adults in good approximation, although we found weak and noisy signals of species properties related to seed dispersal. We hypothesize that spatial mechanisms with strong stochastic components such as animal seed dispersal overpower the pattern-forming effects of dispersal limitation, density dependence and habitat association, or that some of the pattern-forming processes cancel out each other.

## 1. Introduction

Plants can only move during their seed stage; once germinated, they cannot escape interactions with their biotic and abiotic neighbourhood. Understanding the spatial association between adult plants and their offspring is therefore critical to get insights on plant population and community dynamics [1]. However, the different stages from seed dispersal to the sapling stage are often difficult to study, especially in tropical forests. For example, seed limitation in tropical forests is pervasive [2], and seed arrival of most tropical tree species is sparse and patchy [3]. The template generated by primary seed dispersal is then modified by secondary seed dispersal [4], post-dispersal processes such as seed predation [5], microhabitat requirements for establishment [6] and density-dependent survival [7]. The spatial adult–offspring association, in turn, can be relatively easily studied in large plots of fully mapped forest communities [8] such as those sampled within the network of the Center for Tropical Forest Science (CTFS) [9,10]. This association constitutes the (medium-term) accumulated outcome of all processes and mechanisms from seed production at the parent tree up to the point where the offspring has reached a certain size threshold (e.g. 1 cm diameter at breast height to enter the census called in the following ‘recruits’). We therefore expect that the spatial adult–recruit association should conserve imprints of past processes and be a useful ‘ecological archive’ from which we may obtain hints on the relative importance of underlying processes and the type of population and community dynamics [8,11].

Various mechanisms have the potential to result in distinct spatial associations between adults and conspecific recruits. Our *a priori* hypothesis is that recruits should be positively associated with adults because of dispersal limitation [12,13] where seeds are dispersed in their majority close to their parents. However, even if a species shows strong dispersal limitation, the resulting adult–recruit association may be more complex because recruits (which are mostly saplings) can stay for decades below sizes of 1 cm diameter at breast height (DBH) waiting for conditions becoming favourable for growing [14]. In this case, a substantial proportion of their parents may have already died and we may rather observe association patterns of the ‘partial overlap’ type [15–17], where recruits aggregate around parents that are still alive, but recruits without living parents are locally segregated from the current adults. Similar patterns of partial overlap can also emerge under dispersal limitation if species exhibit intraspecific spatial variation in seed production. However, seed dispersal by bats, non-volant mammals, birds or wind can contribute to independent or segregated adult recruit associations. For example, the behaviour of large frugivores that defecate seeds in masses, or central-place foragers that move seeds from a wide area to nests, may cause aggregated recruit patterns [18] that are largely decoupled or spatially independent from the locations of the parent trees [8]. By contrast, wind-dispersed species may show less likely seedling clumping than animal-dispersed species [3], making spatially segregated adult–recruit patterns more probable. Another prominent mechanism that has the potential to generate positive adult–recruit associations is association of species with a certain topographic habitat feature such as slope [19,20] or soil type [21,22]. However, if particular associations with environmental covariates change from recruits to adults [20,23,24], this can generate partial overlap or segregation patterns.

Finally, although dispersal limitation [13] or shared habitat association lets us expect a close adult–recruit association, propagules falling close to fruiting adults only rarely produce saplings because species-specific predators and pathogens make the direct neighbourhood of a parent tree inhospitable for the survival of seedlings. This mechanism, known as Janzen–Connell effect [25,26], or negative plant–soil feedback mediated by soil biota [27], can counteract positive associations at smaller neighbourhood scales and may lead to independence or segregation [13]. It is clear, however, that the processes and mechanisms described above will each operate at distinct spatial scales and that an analysis of adult–recruit association therefore needs to explicitly account for spatial scale. Thus, habitat associations, seed dispersal and negative density dependence have strong potential to generate distinct scale-dependent spatial association patterns in the adult–recruit relationship.

Recent advances in spatial point pattern analysis [28,29] combined with the availability of large fully mapped plots of tropical forest [9,10] have produced an inspiring new perspective on the structure of hyperdiverse communities [15–17] and allow us to quantify adult–recruit patterns for a large number of species even in species-rich forests. This provides unique opportunities for testing whether positive adult–recruit associations do indeed prevail and for deriving hypotheses on the relative importance and the interplay of the various processes and mechanisms listed above. The spatial association between the adult and recruit generation also has important consequences for the type of community dynamics and species coexistence. For example, strong positive spatial associations, where recruits are tightly clustered around the adult trees, yield intraspecific aggregation and interspecific segregation, which can enhance the local coexistence in plant communities by increasing the importance of intraspecific competition relative to interspecific competition [30,31].

A fundamental first task in quantifying the spatial adult–recruit association is testing for statistical independence between the spatial distribution of the recruits from that of the adults and quantifying the magnitude of departures from independence. If this null hypothesis is rejected, we can then continue to quantify the spatial association patterns in more detail [32] and relate it to species properties. However, as already noted by Lotwick & Silverman [33], implementing a null model of independence is complicated because it must break the possible spatial association between the two patterns while conserving the statistical properties of the observed univariate spatial patterns [34], such as the observed spatial autocorrelation [33].

In this study, we used techniques of spatial point pattern analysis [17,28,29] to quantify the intraspecific adult–recruit associations for more than 60 species over six censuses in the 50 ha forest dynamics plot on Barro Colorado Island (BCI), Panama. We structured our analysis into three parts. First, patterns generated by pattern reconstruction [35,36] served as null expectation to test whether recruits are spatially independent from the adults. Second, we updated the classification scheme developed by Wiegand *et al.* [17,32] to more closely characterize the magnitude and types of potential departures from independence at different neighbourhood scales around adult trees. Finally, we tested for statistical relationships between the type of adult–recruit association and species properties such as shared associations to topographic habitat variables, shade-tolerance guild or dispersal mode. We used the results of our comprehensive analysis to derive hypotheses on the relative importance of processes that determine the spatial distribution patterns of recruits and discuss the consequences of our findings for population and community dynamics.

## 2. Material and methods

### (a) Study area

The tropical forest at BCI, Panama (9°10′ N, 79°51′ W) is a seasonally moist tropical forest that hosts more than 300 tree and shrub species. Rainfall averages 2600 mm yr^−1^, with a pronounced dry season. Investigations were carried out with data from the 50 ha forest dynamics plot, which consists of mainly old-growth lowland moist forest. Elevation ranges from 120 to 155 m above mean sea level. The plot was established in 1982 and all trees with at least 1 cm DBH have been mapped, tagged and measured every 5 years since 1985. Details on the plot are provided by Condit [9] and Losos & Leigh [10].

We used the data from the six 1982, 1985–2010 census combinations [37]. We included all living trees, but excluded shrub and liana species, and divided them into the two non-overlapping life stages recruits and adult trees. Adults were defined based on a species-specific DBH threshold for reproductive size provided by Hubbell *et al.* [37], and recruits were all trees that entered the census for the first time (i.e. crossed the 1 cm DBH threshold during the last 5 years). To compare the patterns of recruits with those of their potential parent trees, we used the adult trees from the previous census. To obtain sufficient sample sizes, we restricted our analysis for a given census to species that had at least 50 adults and at least 50 recruits. We obtained for the different census periods datasets for 40–53 different species, representing a total of 65 species.

### (b) Testing for independence between adults and recruits (analysis 1)

To detect potential association patterns in the placement of recruits around adult trees, we used the null model of independence [29]. Because the adults are antecedent to the recruits, we keep the adult locations fixed but randomize the locations of the recruits. To conserve the spatial autocorrelation structure [33,34] in the independence test, we used non-parametric techniques of pattern reconstruction to create stochastic replicates of the observed recruit patterns that are used in the null model [29,35,36]. Pattern reconstruction uses optimization techniques (for details, see the electronic supplementary material, appendix A) to reconstruct a point pattern based on the information provided by several summary statistics calculated from the observed pattern [36,38]. The spatial structure of the reconstructed patterns very closely matches that of the observed patterns.

To evaluate departures between the observed data and the stochastic realizations of the null model, we need summary statistics that are able to quantify the observed and simulated spatial patterns. Illian *et al.* [39] and Wiegand *et al.* [36] recommend using several summary statistics of different nature to test for potential departures from a null model. This is especially important for ‘real-world’ patterns, which often show aspects of heterogeneity (see below). Here, we use the bivariate *K*-function *K*_12_(*r*) and the bivariate distribution function of the nearest-neighbour distances *D*_12_(*r*) [39] for this purpose. The *K*_12_(*r*) can be defined as the mean number of recruits within neighbourhoods with radius *r* around the adult trees divided by the mean density *λ*_2_ of recruits in the plot, but in analysis 1 we used the transformation *L*_12_(*r*) = (*K*_12_(*r*)/*π*)^0.5^ – *r* to stabilize the variance [33]. The *D*_12_(*r*) gives the proportion of adult trees that have at least one recruit within distance *r*.

We estimated simulation envelopes from 199 simulations of the independence null model. A departure from the null model at a given neighbourhood distance *r* around adult trees was indicated if a given summary statistic of the observed data (i.e. *D*_12_(*r*) or *K*_12_(*r*)) was outside the simulation envelopes being the fifth lowest and highest values of the summary statistic estimated from the simulations of the null model. However, because the simulation envelope test is prone to type I error inflation, we used a goodness-of-fit test (GoF) [40] to determine the overall fit of the null model over the distance interval 1–250 m. Significant departure occurred if the GoF was significant with a 2.5% error rate for at least one of the two summary statistics (in the ‘worst’ case of independence of the two summary statistics this yields a joint error rate of ≈5%).

### (c) The type of the adult–recruit association pattern (analysis 2)

The GoF test provides only a binary assessment of the spatial adult–recruit association and does not convey information on the magnitude of effects and the type of departures. The goals of this analysis were therefore to determine how the recruits (pattern 2) of a given species were distributed within neighbourhoods of conspecific adults (pattern 1), and how strongly they departed from independence. In case of homogeneous patterns (i.e. the spatial configuration of recruits around adults is the same all over the plot and only subject to stochastic fluctuation, which follows the same laws within the entire plot [39]), we would have only two possible types of departure attraction and segregation, and departures in *D*_12_(*r*) and *K*_12_(*r*) would be highly correlated. However, the spatial configuration of recruits around adults may show high variability and spatial trends. For example, some adults may have many neighbouring recruits but others very few. In this case, *D*_12_(*r*) and *K*_12_(*r*) will show contrasting results [29,32]. We therefore updated the classification scheme developed by Wiegand *et al.* [32] and used the standardized effect sizes of *D*_12_(*r*) and *K*_12_(*r*) to classify the type and strength of departures from independence2.1
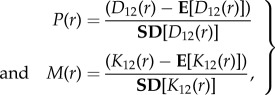
where the operators **E**[*.*] and **SD**[.] indicate the expectation and standard deviation of the summary statistic at neighbourhood *r* under independence, respectively.

**Figure 1.**
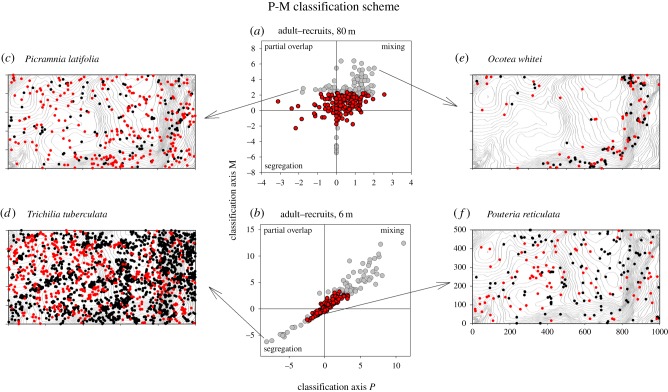
Classification of adult–recruit associations at the 1000 m × 500 m BCI forest dynamics plot. (*a*) Allocation of the adult–recruit associations of all analysed species for all six censuses and a large-scale neighbourhood radius of *r* = 80 m (significant cases: grey circles; non-significant cases: red circles). (*b*) The same as (*a*) but for a small-scale neighbourhood of *r* = 6 m. Axis *P* is positive (negative) if there are on average more (fewer) recruits at the scale *r* from adults than expected, and axis *M* is positive (negative) if the probability that an adult has its nearest recruit neighbour within distance *r* is larger (smaller) than expected. (*c*) Example for partial overlap at large scale (red circles, adults; black circles, recruits) of the species *Picramnia latifolia.* (*d*) Example for small-scale segregation of the species *Trichilia tuberculata*. (*e*) The large-scale mixing of *Ocotea whitei* reflects their consistent habitat association to covariates such as slope and TWI. (*f*) Example of a species whose adult–recruit association cannot be distinguished from independence at small scales. The grey contour lines in the back of the four maps show the elevation of the BCI plot.

The location of each species within the two-dimensional classification space (figure 1*a*,*b*) conveys information on the significance and strength of the adult–recruit relationship. Because the distribution of *P*(*r*) and *M*(*r*) is approximately the standard normal distribution, the box delimited by values of −2.33, 2.33 (which correspond to a *p*-value of 0.025 for two summary statistics individually) approximates the area where the null hypothesis cannot be rejected, and a given species departs more strongly from independence the farther away it is located from the box. However, the quadrant of the scheme where the species is located provides additional information on the type of departure. In addition to independence, four other types of spatial association patterns are possible for each neighbourhood *r* [32]:

— **Type 0**: ‘**independence**’: neither *K*_12_(*r*) nor *D*_12_(*r*) show significant departures from independence (figure 1*f*).
— **Type I**: ‘**segregation**’: recruits occur consistently less within neighbourhoods with radius *r* around adult trees than expected under independence (*M*(*r*) < 0, *P*(*r*) < 0; figure 1*d*).
— **Type II**: ‘**partial overlap**’: recruits occur on average more often within the neighbourhoods of adult trees than expected [*M*(*r*) ≥ 0], but a notable proportion of adult trees have less nearest recruit neighbours than expected (*P*(*r*) < 0; figure 1*c*). This type occurs only for heterogeneous patterns.
— **Type III**: ‘**mixing**’: recruits occur consistently more often within the neighbourhood of adult trees than expected (*M*(*r*) ≥ 0, *P*(*r*) ≥ 0; figure 1*e*).
— **Type IV**: This association type corresponds to (*M*(*r*) < 0, *P*(*r*) ≥ 0) and is predicted to occur only rarely [32] (if adult trees are highly clustered and few recruits are close to the adult clusters).

To explore how the proportion of these association types changed with the neighbourhood *r* around the adult trees, we used all species for which the GoF test indicated significant departures from independence, and counted for each association type and neighbourhood radius *r* the number of cases where the observed value of the summary statistic was located outside the simulation envelopes [17]. All other cases were added to the independence type.

### (d) Relationships between association type and species properties (analysis 3)

In case that analysis 1 indicated departures from independence, we were interested to find hints on potentially underlying mechanisms to facilitate formulation of hypotheses. An obvious hypothesis for positive associations (i.e. mixing or partial overlap) is shared habitat association. Several methods exist to test for this, with the most popular being a torus translation test [19] where associations of a species pattern with *a priori* defined discrete habitat types is investigated. However, we used here the Berman test [41], which tests association to continuous topographic habitat variables. Topographic variables are considered good surrogates for unavailable more direct environmental variables and were successful in detecting species assemblages at the BCI forest that agreed [20] with previous assumptions [19]. We used here the six spatial covariates of Kanagaraj *et al.* [20]: elevation (Elv), slope (S), aspect (Asp), convexity (Con), topographical wetness index (TWI) and vertical distance to streams (VDS) at a spatial resolution of 5 m × 5 m quadrats.

We used the Berman test [41] to test for spatial independence of the recruit and adult pattern of the 65 species from the six (continuous) topographic covariates. The Berman test is based on the mean *S*_obs_ of the covariate values *v*(**x**_i_) at the locations **x**_i_ of the trees of a given pattern, which are then compared with the corresponding *S*_sim_ values obtained from repeated simulations of a suitable null model. Significant departures from the null model are assessed by the test statistic *Z*_1_ = (*S*_obs_−*μ*)/*σ*, where *μ* is the mean value of *S*_sim_ under the null model and *σ*^2^ the corresponding variance [41]*.* The null distribution of this test statistic is approximately the standard normal distribution. To consider the spatial autocorrelation (clustering) of the recruit and adult patterns [41], we used the null model of pattern reconstruction as applied in analysis 1 (for details, see the electronic supplementary material, appendix B).

**Figure 2.**
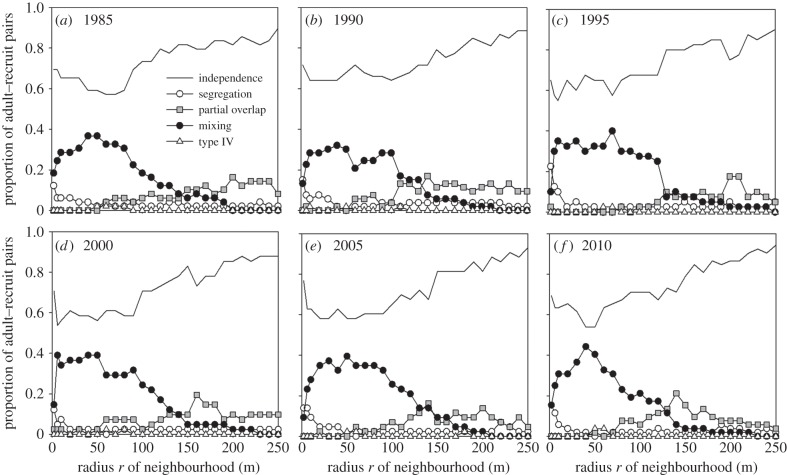
(*a*–*f*) Spatial pattern analysis of adult–recruit associations and their scale-dependent changes. The most common type was ‘independence’ between adults and recruits, followed by ‘mixing’, ‘partial overlap’ and ‘segregation’.

To test whether the five association types were statistically related with species properties, we used a permutation test proposed by Hothorn *et al.* [42]. We conducted the permutation test separately for small (i.e. 2–10 m) and large neighbourhoods (60–100 m). We selected these distances because they represent neighbourhoods where deviations from independence were most interesting (i.e. segregation peaked at smallest scales while mixing peaked between 60 and 100 m; cf. figure 2) and because they reflect contrasting scales of direct tree–tree interaction effects at small and influences from habitat at large scales. The permutation test reveals whether the association type depended statistically on the individual categories of the life traits ‘shade-tolerance guild’ and ‘dispersal agent’ (for details, see the electronic supplementary material, appendix C, and tables C1 and C2).

We approximated the null distribution of independence of association between the categorical data based on Monte Carlo re-sampling techniques with 10 000 permutations to assess *H*_0_ at *α* = 0.05, and determined the *maxT*-statistic and the associated *p*-value [42]. Finally, we also present a standardized linear statistic that can be interpreted similarly to Pearson residuals for the independence hypothesis [42]. In this standardized contingency table, large positive or negative values highlight deviation from independence in favour of a positive or negative association between both variables, respectively. The permutation tests were executed with the R software package *coin* [42], whereas the spatial pattern analysis was done with the software Programita [29] (which can be accessed at www.Programita.org).

## 3. Results

### (a) Testing for independence between adults and recruits (analysis 1)

The GoF test over the 1–250 m distance interval revealed that the proportion of species with independent adult–recruit association made up 71.4%, 54.7%, 70.0%, 61.0%, 69.8% and 71.2% of the adult–recruit pairs in the 1985–2010 censuses, respectively (66.4% for all censuses combined). However, if we consider the proportion of independence for individual neighbourhood radii (i.e. cases where the species had a non-significant GoF test, or both summary statistics were inside the simulation envelope at neighbourhood *r*), adult–recruit pairs showed a peak of independence at the smallest neighbourhood (2 m) and a steady increase towards larger scales from approximately 100 m onwards (figure 2). Averaged over all spatial scales 1–250 m and summarized for all six censuses, independence was the most common type, which accounted on average for 73.2% of all cases.

Independence between recruits and adults was little affected by temporal variation in maturation or senescence of the potential parent cohort. GoF tests for independence of recruits of the 2010 census and adults of the six earlier censuses revealed independence for 63.3–73.3% of the adult–recruit pairs (see the electronic supplementary material, appendix D and figure D1).

### (b) The type of the adult–recruit association pattern (analysis 2)

Averaged over all 1–250 m neighbourhoods, and summarized for all six censuses, ‘mixing’ and ‘partial overlap’ were, with 16.4% and 6.5%, the second and third most common association types, respectively. Mixing mostly dominated the significant deviations from independence at the first 130 m, having its peak at large scales between 60 and 100 m (figure 2). ‘Segregation’ occurred on average only in 3.6% of all cases. The plots of the *P*–*M* scheme showed that although there are a few outlier species, most of the species fall within or close to expectation under independence (figure 1*a*,*b*).

### (c) Relationships between association type and species properties (analysis 3)

Analysis with the Berman test revealed that the dependency of the species distribution patterns on topographic covariates was relatively weak (electronic supplementary material, figure E1 and appendix E). We also found that shared habitat association of adults and recruits occurred at small neighbourhoods (2, 6 and 10 m) for 23–46% (average 36.6%) of the species that showed mixing (electronic supplementary material, appendix E). The same analysis was also done for large neighbourhoods where mixing peaked (60, 80 and 100 m; figure 2). We found in 27–57% (average 44.3%) of the cases consistent association of adults and recruits to the topographic covariates may have caused the mixing at large scales (electronic supplementary material, appendix E). Thus, even though topography had stronger effects on the positive association type, it contributed to adult–recruit mixing in fewer than half of the cases.

The permutation test suggested relationships between life-history strategies and the spatial association pattern of recruits around adults. According to the large positive values of the contingency table, shade-tolerant species (S) tended to show at small scales only segregation but no mixing or partial overlap, whereas gap species (G) tended to show no segregation but partial overlap and mixing (table 1*a*). At larger scales, shade-tolerant species tended to show also segregation but no independence (table 1*a*). Individual dispersal agents showed mostly weak or inconsistent relationships with spatial association types (electronic supplementary material, appendix C and table C2). However, the permutation tests for a single category (comprising bats, birds and mammals) revealed at small scales the highly significant result that animal-dispersed species are positively related to independence, species with explosive seed dispersal to mixing (but not independence) and wind-dispersed species to segregation (table 1*b*). At large scales, effects were relatively weak.

**Table 1.** Results of the permutation tests of independence between life-history strategies and spatial patterns at small- (2–10 m) and large-scale (60–100 m) distance intervals. *p* < 0.05 indicates that the four types of adult–recruit association depend on the life-history strategies. Large positive or negative values in the standardized contingency table highlight deviation from independence in favour of a positive or negative association between spatial patterns and the individual categories of the life traits (left column): light demanding gap specialist (G), intermediate (I) and shade-tolerant (S) species, animal, explosive (Exp) and wind-dispersed species. For details, see Results section and electronic supplementary material, appendix C.

	independence	segregation	partial overlap	mixing	independence	segregation	partial overlap	mixing
(*a*)	shade-tolerance guild: small scale				shade-tolerance guild: large scale			
G	0.01	−3.32	1.52	1.53	1.72	−1.73	−0.94	−0.84
I	0.82	−0.53	−0.67	−0.52	2.33	−1.22	−1.34	−1.47
S	−0.54	3.17	−0.85	−0.96	−2.97	2.26	1.67	1.67
*maxT* = 3.3239, *p* = 0.0156					*maxT* = 2.9715, *p* = 0.035			
(*b*)	dispersal agent: small scale				dispersal agent: large scale			
animal	5.88	−7.13	−1.45	−1.20	−2.99	0.96	1.52	2.16
Exp	−4.36	−1.48	2.75	5.51	0.11	−0.60	−0.95	0.52
wind	−3.93	10.32	−0.41	−2.99	3.76	−0.73	−1.16	−3.21
*maxT* = 10.3226, *p* < 2.2 × 10^−6^					*maxT* = 3.7559, *p* = 0.0068			

## 4. Discussion

In this study, we conducted a comprehensive analysis of the spatial association of newly recruited saplings at the BCI forest relative to their potential parents. We found that the spatial pattern of recruits was in approximately three-quarters of the cases independent from the adult trees, but showed in 16% of the cases the expected positive associations, in 7% partial overlap, and only in 4% segregation. The remarkable prevalence of independence between recruit and adult patterns can mean that (i) there is real independence, (ii) stochastic effects overpower a potential biological signal in the data or (iii) the test is not sensitive enough. We reduced the risk of (iii) by using the pattern reconstruction implementation of independence and by using two summary statistics simultaneously. Option (i) is in contrast to expectations from many of the distance-dependent processes that are known to operate in tropical forests and have a strong potential to create smaller-scale (i.e. less than 50 m) association patterns during the transition from seeds to recruits. We expected that dispersal limitation [2,13], one of the cornerstones of neutral theory [43], should result for most species in smaller-scale positive associations where recruits are clustered around adults. Additionally, shared or opposed habitat association should result in positive or negative small- or larger-scale association, respectively, and Janzen–Connell effects should result in smaller-scale segregation between recruits and adults [5,19,23].

Our first analysis revealed that an overwhelming majority of species showed independence between adults and recruits. We therefore could not expect to find in our subsequent analyses strong relationships between association types and species properties. Although shared habitat association of recruit and adult trees is a candidate mechanism for creating positive ‘mixing’ association, we found that fewer than half of the species with mixing showed consistent associations to topographic habitat variables for both adults and recruits. Additionally, we confirmed earlier studies showing that habitat preference changed often between different life stages within the same species so that over time recruits or adults lost or attained habitat dependency [20,23]. The particularly strong fluctuations in habitat association of the recruit communities over the six censuses (electronic supplementary material, figure E1a) suggest that potential habitat associations were strongly masked by unpredictable chance events in dispersal (i.e. stochasticity; option (ii)). In contrast to a recent study by Baldeck *et al.* [44] that was based on the proportion of community compositional variation in fixed scale 20 m × 20 m subplots, the significances of the habitat associations in our study were little dependent on sample size (see the electronic supplementary material, appendix E and table E).

The hypothesis on the strong stochastic component of dispersal (option (ii)) is supported by our permutation test (table 1), which showed that animal-dispersed species tended to show independence. Most of the 65 study species are dispersed by animals, but BCI hosts 24 species of non-volant mammals, 20 species of bats and 86 species of birds that may jointly disperse seeds of one and the same species [3]. While individual dispersal agents show (rather weak) relationships with spatial association types (see the electronic supplementary material, appendix C and table C2), the joint outcome of differences in spatial movement behaviour of these taxa has the potential to create strong stochasticity in the resulting dispersal patterns that diffuses deterministic adult–recruit associations. While some of this stochasticity may be reduced by more detailed data and more refined models of disperser behaviour, a substantial proportion of variability will remain due to the inherent stochasticity of the complex dispersal process [45,46], similar to long-term weather forecasts. We hypothesize that this complexity is a major cause of the surprising dominance of independent associations between adults and recruits on BCI.

An alternative explanation for the high incidence of independence found here is that some of the pattern-forming processes cancel out each other (i.e. option (i)). For example, highly aggregated seed distributions typical of dispersal kernels [3,47] may be removed by Janzen–Connell effects or more general density-dependent mortality. This is in accordance with recent findings by Terborgh *et al.* [13] and others [48] that showed that undispersed seeds (i.e. seeds that fell beneath the crown of conspecific fruiting adults) contribute little or nothing to sapling recruitment and that newly recruited saplings occurred at locations independent from that of reproductive conspecifics. Another explanation for the high incidence of independence would be that recruits emerged only decades after seed dispersal and that most of their parents had already died. However, we could reject this hypothesis at least for a 30-year interval when comparing the recruits of the last census with the adults of all earlier censuses (see the electronic supplementary material, appendix D and figure D1). Clearly, independence could also be a result of lack of analytical power of our method (option (iii)) because the datasets for some very few species are based on 50 adults and 50 recruits. However, we found that significant adult–recruit associations did not primarily depend on the sample sizes, although significant effects tended to be slightly more frequent for larger sample sizes at the smallest neighbourhood of *r* = 2 m (see the electronic supplementary material, appendix F and table F4). Additionally, the BCI plot provides already an incredibly large dataset for our analysis and if we cannot statistically detect more significant effects in this dataset, we have to conclude from a practical standpoint that stochastic effects overpower deterministic effects at the BCI forest, if the latter were present to a large extent (i.e. option (ii)). This is our main hypothesis for the observed lack of spatial dependence between recruits and adults. As outlined above, the highly stochastic animal dispersal process is a promising candidate mechanism for overpowering deterministic spatial structure. The high incidence of statistically independent adult–recruit associations is an intriguing result that requires an explanation, but we have to leave the more detailed search for the underlying mechanisms to further studies. The question of whether strong pattern-forming processes and mechanisms cancel out each other or are overpowered by stochastic processes has important implications for our understanding of the dynamics of species-rich forests.

## 5. Conclusion

We found that the outcome of the multiple and complex processes that determine the location and survival of recruits resulted in the majority of cases in spatial independence between recruits and their potential parents, and that habitat and species traits left in relatively few cases a detectable signal of non-independence. What are the consequences of our findings for long-term dynamics and coexistence in diverse tropical forests? Overall, it is interesting to see that the cumulative outcome of several directed processes results in associations that mostly cannot be distinguished statistically from independence. That means that the recruit community is in space largely decoupled from the adult community. Although it may be tempting to interpret the independent adult–recruit relationship in favour of neutral theory, such a conclusion is premature because neutral theory does not explicitly consider a recruit community [43] and therefore cannot be directly compared with our analysis.

The finding that the recruit community is spatially decoupled from the adult community means that the forest will remain diverse in the long term because the next adult generation is likely to be placed at somewhat different locations than the current one. This is a mechanism that avoids high local densities of particular species and therefore acts, similarly to Janzen–Connell effects, as a stabilizing mechanism [49]. Independent placement of offspring relative to the parents may prevent species from developing, over evolutionary time, distinctive life-history strategies, because at the end stochasticity determines the final survival location [50]. Indeed, most species on BCI have not extreme but intermediate light requirements and lifestyles [51].

Our results suggest several avenues for future work. Repeating the analyses for forests where animal seed dispersal is less important or where dispersers went extinct [52] would enable researchers to test the hypothesis of stochastic animal dispersal, and it would be important to include forests that show a larger degree of environmental heterogeneity than the BCI plot. Finally, conducting analyses for forests with different species richness can show whether prevalence of independence is related to species richness (i.e. the dilution hypothesis [17,53]). Assessing what is different in forests with different prevalence in spatial patterns should allow us to reach a broader understanding of the relative importance of processes for the adult–recruit relationship and forest dynamics.

## Supplementary Material

Electronic supplementary material - ESM
