# Supplementary material for: Stochastically driven adult–recruit associations of tree species on Barro Colorado Island
Source: Proc Biol Sci. 2014 Sep 7;281(1790):20140922. doi: 10.1098/rspb.2014.0922 (PMC4123702; doi:10.1098/rspb.2014.0922)
Supplement: Electronic supplementary material - ESM [file rspb20140922supp1.doc]

**Electronic supplementary material - ESM**

***Appendix A:*** *Pattern reconstruction algorithm for independence null model*

Implementing a null model of independence is a highly nontrivial task because it requires a null model that breaks the possible spatial association between the two patterns while conserving the univariate spatial pattern of the species. If we fix the focal pattern (i.e., the antecedent adult pattern) the problem is reduced to the task of generating replicates of the observed recruit pattern that maintain the univariate characteristics of the pattern (e.g., number of points and the observed spatial autocorrelation in the placement of individuals) but are statistically independent from the focal pattern of adults. Because the spatial pattern of a species is in general the outcome of stochastic processes, the null model should not produce exact copies of the spatial univariate pattern but stochastic replicate patterns that show the same properties as the observed pattern.

Thus, we need to generate recruit patterns that show the same stochastic characteristics as the observed recruit pattern. Clearly, using the homogeneous Poisson process (i.e., a completely random pattern) as null model is only valid if the species pattern does not show small-scale aggregation (i.e., no autocorrelation). This can be easily understood. For example, if both patterns show aggregation it may happen quite frequently that the clusters of the two patterns largely overlap just by chance, or segregate just by chance. However, it is unlikely that such configurations may arise under the homogeneous Poisson process (because it does not conserve aggregation) and as a consequence, it may indicate spurious attraction and segregation, respectively.

One approach to overcome this problem is the toroidal shift (Lotwick and Silverman 1982; Berman 1986, Harms et al. 2001; Wiegand and Moloney 2004), which produces replicate patterns that approximately maintain the observed univariate spatial structure. In this null model, the species pattern is shifted in its entirety by first adding a fixed random vector (dx, dy) to each coordinate and then re-assembling the shifted pattern by wrapping it on a torus. However, this approach can create artifacts by arbitrarily connecting and interrupting parts of the continuous structures of the pattern, and it does not really produce a stochastic replicate of the observed pattern because most of the point-point distances are maintained. A second approach to generate stochastic replicate patterns is to fit parametric point process models to the species pattern and use the realizations of the fitted model as null model (Plotkin et al. 2000; John et al. 2007). Because the major concern is small-scale clustering, the Thomas process (Wiegand et al. 2007) has been used mostly for this purpose. It represents the simplest case of a point process model that can generate clustered patterns and has the convenient property that the analytical form of its pair correlation function is known. This allows fitting its parameters to an observed pattern. However, the Thomas process captures only one critical scale of clustering (Wiegand et al. 2009) and only the second-order feature of a point pattern (i.e., the pair correlation function and *K*-function). However, it is well known that several different cluster processes may show the same pair correlation function but different nearest neighbour summary statistics (Tscheschel and Stoyan 2006; Wiegand et al. 2007; Wiegand et al. 2013). This means that generating the patterns with a Thomas process may miss out important features of the spatial structure of the observed pattern and therefore yield an incorrect representation of essential aspects of the univariate spatial pattern. Additionally, the pair correlation function (and the *K*-function) captures only some aspects of the potentially complex spatial structure of real world spatial patterns (Wiegand et al. 2009, 2013) which may render parametric fits as an unsuitable approach. These problems can be partly solved by using more complex point process models which are able to mimic more complex spatial structures (e.g., Wiegand et al. 2009), but this approach is severely limited by the technical problems associated with fitting several summary statistics simultaneously to the observed pattern.

In this study we use non-parametric techniques of pattern reconstruction (Tscheschel and Stoyan 2006; Illian et al. 2008; Wiegand et al. 2013) as solution to the problem of generating species patterns that show the same stochastic characteristics as the observed pattern. To this end we used the pattern reconstruction algorithm described in Wiegand et al. (2013) that is based on methods presented in Tscheschel and Stoyan (2006). This algorithm is a variation of simulated annealing (Kirkpatrick et al. 1983) that generates by trial and error a series of patterns that approach in each simulation step the summary statistics of the observed patterns more closely. The statistical properties of the observed pattern *φ* are measured by several functional summary statistics $f_{i}^{\phi}(x)$ where the variable *x* may represent distance *r*. During each simulation step *t* we estimated the corresponding summary statistics $f_{i}^{\psi_{t}}(x)$ of the simulated pattern *ψ_t_* to estimate the deviations

(1)

between$f_{i}^{\phi}(x)$and $f_{i}^{\psi_{t}}(x)$ where the variable *x* is evaluated at *n*_i_ discrete values *x*_b_. To combine the deviations *E^φ^_i_* arising from different summary statistics *i* into a total deviation measure we need to normalize with weights *w*_i_ in a way that the different summary statistics yielded approximately the same value of *E^φ^_i_* if the observed and simulated patterns approached a good agreement (for details see Wiegand et al. 2013). The total deviation yields:

$E_{_{total}}^{\phi}(\psi_{t})=\frac{\sum_{i=1}^{I} w_{i}E_{i}^{\phi}(\psi_{t})}{\sum_{i=1}^{I} w_{i}}$ (2)

The reconstruction of pattern *φ* starts with a random pattern *ψ*_0_ that has the same number of points as *φ*. In each simulation step *t* a randomly selected point is tentatively removed and a new point with random coordinates is proposed instead. This new point is accepted if $E_{_{total}}^{\phi}(\psi_{t})<E_{_{total}}^{\phi}(\psi_{t-1})$, otherwise another new point is considered (Tscheschel and Stoyan 2006; Wiegand et al. 2013). Thus, the new pattern *ψ*_t_ is slightly more similar to the observed pattern than the previous pattern *ψ*_t-1_. This algorithm is able to find local minima with very small total deviation (Wiegand et al. 2013) and each simulation will end up in a different local minimum (the absolute minimum would be the observed pattern *φ*). The pattern reconstruction algorithm therefore generates the stochastic replicates of the observed pattern required for the independence null model.

***Appendix B:*** *Berman test of association with spatial covariates*

We used the Berman test (Berman 1986) to investigate if the density of adult or recruit patterns was dependent on any of the six continuous topographic covariates. In the conventional Berman test, the observed distribution of the values of a spatial covariate *Z* at the tree data points *x* and the predicted distribution of the same values under the null model of complete spatial randomness (CSR) are compared using the *Z*_1_ test statistic. *Z*_1_ is computed based on the sum *S* of the covariate values at all data points. The predicted mean *µ* and variance *σ*^2^ of *S* are then computed from the values of the covariate at all locations in the window. Then *Z*_1_ = (*S*‑*µ*)/*σ.* Based on this test statistic one can formulate the null and alternative hypotheses. *H*_0_: *X* is a stationary Poisson point process independent of *Z*. *H*_1_: conditionally on *Z*, the process *X* is an inhomogeneous Poisson point process with intensity depending on the distance from *Z*.

However, this test does not take into account the effect of spatial autocorrelation (clustering) in the spatial pattern of recruits and adults and provides therefore a non-precise estimate of habitat association (Berman 1986). For example, if the species pattern is clustered and a cluster accidently overlaps a patch with a high or low value of the covariate, a null model that does not maintain the observed clustering may indicate a significant association because this configuration will be unlikely under this null model. However, such a configuration may appear just by chance under a null model that conserves the observed clustering. We therefore generated null distributions of the observed species pattern based on the non-parametric technique of pattern reconstruction (Appendix A above) that is able to generate stochastic replicates of the observed pattern that approximate several summary statistics of the observed pattern very well (i.e., pair correlation function, *K*-function, spherical contact distribution, nearest neighbour distribution functions). We assessed significant deviation of the *Z*_1_ values from *H*_0_ at α = 0.05.

***Appendix C:*** *Species properties and permutation test*

*Permutation test*

Details on the permutation test are provided in Hothorn et al. 2006. The non-parametric permutation tests were based on linear associations between the three to five individual categories of the life traits as independent variable and the association patterns independence, segregation, partial overlap, and mixing as dependent variables. To get a meaningful data basis, we lumped for each permutation test all species from all six censuses and from the three distances 2m, 6m, 10m (small scale test) and 60m, 80m, 100m (large scale test). Treating the three joint distance intervals as so-called block factor (Hothorn et al. 2006) increases the test power because adult-recruit associations should be relatively similar within each of the small- and large-scale classes.

*Species properties*

The shade-tolerance guilds were defined as the three categories: light demanding gap specialist (G), intermediate (I), and shade tolerant (S) species, based on the classification of Comita et al. (2007), as well as other literature (e.g. Beckman and Muller-Landau 2007; Goldsmith and Zahwahi 2007). The dominant dispersal agent was identified for each species based on information from Dalling et al. (2002), Muller-Landau et al. (2008), and the online-data base of Wright (2011). We assigned a total of five predominant classes of dispersal modes to the species: bat (Bat), bird (Bird), explosive (Exp), mammal (Mam), and wind (Wind). Following Muller-Landau et al. (2008), mammals are distinguished from bats as being non-volant mammals. Information on all species properties are compiled in Table C1.

***Table C1:*** *Information on the 65 analysed species*

| **species code** | **genus** | **species** | **family** | **shade tolerance guild** | **dispersal agent** |
| --- | --- | --- | --- | --- | --- |
| ALCHCO | *Alchornea* | *costaricensis* | Euphorbiaceae | G | Bird |
| ALIBED | *Alibertia* | *edulis* | Rubiaceae | S | Mam |
| ALSEBL | *Alseis* | *blackiana* | Rubiaceae | S | Wind |
| APEIME | *Apeiba* | *aspera* | Tiliaceae | G | Mam |
| BEILPE | *Beilschmiedia* | *pendula* | Lauraceae | I | Mam |
| BROSAL | *Brosimum* | *alicastrum* | Moraceae | I | Bat |
| CASEAC | *Casearia* | *aculeata* | Flacourtiaceae | S | Bird |
| CASSEL | *Cassipourea* | *elliptica* | Rhizophoraceae | S | Bird |
| CECRIN | *Cecropia* | *insignis* | Cecropiaceae | G | Bird |
| CECROB | *Cecropia* | *obtusifolia* | Cecropiaceae | G | Bird |
| CHA2SC | *Chamguava* | *schippii* | Myrtaceae | S | Mam |
| CORDBI | *Cordia* | *bicolor* | Boraginaceae | G | Bird |
| CORDLA | *Cordia* | *lasiocalyx* | Boraginaceae | S | Bird |
| COU2CU | *Coussarea* | *curvigemmia* | Rubiaceae | S | Bird |
| CROTBI | *Croton* | *billbergianus* | Euphorbiaceae | G | Exp |
| CUPASY | *Cupania* | *sylvatica* | Sapindaceae | S | Bird |
| DES2PA | *Desmopsis* | *panamensis* | Annonaceae | S | Mam |
| DRYPST | *Drypetes* | *standleyi* | Euphorbiaceae | S | Bat |
| ERY2MA | *Erythroxylum* | *macrophyllum* | Erythroxylaceae | S | Bird |
| EUGEGA | *Eugenia* | *galalonensis* | Myrtaceae | S | Bird |
| EUGENE | *Eugenia* | *nesiotica* | Myrtaceae | S | Mam |
| EUGEOE | *Eugenia* | *oerstediana* | Myrtaceae | S | Bird |
| FARAOC | *Faramea* | *occidentalis* | Rubiaceae | S | Mam |
| GAR2IN | *Garcinia* | *intermedia* | Clusiaceae | S | Mam |
| GUARGU | *Guarea* | *guidonia* | Meliaceae | S | Mam |
| GUARSP | *Guarea* | *'fuzzy'* | Meliaceae | S | Mam |
| GUATDU | *Guatteria* | *dumetorum* | Annonaceae | S | Mam |
| HEISCO | *Heisteria* | *concinna* | Olacaceae | S | Bird |
| HERRPU | *Herrania* | *purpurea* | Sterculiaceae | G | Bird |
| HIRTTR | *Hirtella* | *triandra* | Chrysobalanaceae | S | Bird |
| INGAQU | *Inga* | *nobilis* | Fabaceae: Mimos. | S | Mam |
| INGAS1 | *Inga* | *acuminata* | Fabaceae: Mimos. | S | Mam |
| INGAUM | *Inga* | *umbellifera* | Fabaceae:Mimos. | S | Mam |
| JAC1CO | *Jacaranda* | *copaia* | Bignoniaceae | G | Wind |
| LACIAG | *Lacistema* | *aggregatum* | Flacourtiaceae | S | Bird |
| LAETTH | *Laetia* | *thamnia* | Flacourtiaceae | S | Bird |
| LUEHSE | *Luehea* | *seemannii* | Tiliaceae | G | Wind |
| MALMSP | *Mosannona* | *garwoodii* | Annonaceae | S | Bird |
| MAQUCO | *Maquira* | *guianensis* | Moraceae | S | Bird |
| MICOAF | *Miconia* | *affinis* | Melastomataceae | G | Bird |
| MICOAR | *Miconia* | *argentea* | Melastomataceae | G | Bird |
| OCOTCE | *Ocotea* | *cernua* | Lauraceae | S | Mam |
| OCOTWH | *Ocotea* | *whitei* | Lauraceae | S | Mam |
| OENOMA | *Oenocarpus* | *mapora* | Arecaceae | S | Bird |
| PICRLA | *Picramnia* | *latifolia* | Picramniaceae | S | Mam |
| POULAR | *Poulsenia* | *armata* | Moraceae | S | Bat |
| POUTRE | *Pouteria* | *reticulata* | Sapotaceae | S | Bird |
| PRI2CO | *Prioria* | *copaifera* | Fabaceae: Caesal. | S | Mam |
| PROTPA | *Protium* | *panamense* | Burseraceae | S | Bird |
| PROTTE | *Protium* | *tenuifolium* | Burseraceae | S | Bird |
| QUARAS | *Quararibea* | *asterolepis* | Bombacaceae | S | Mam |
| RANDAR | *Randia* | *armata* | Rubiaceae | S | Bird |
| SIMAAM | *Simarouba* | *amara* | Simaroubaceae | I | Mam |
| SIPAPA | *Siparuna* | *pauciflora* | Monimiaceae | S | Bird |
| SOCREX | *Socratea* | *exorrhiza* | Arecaceae | I | Mam |
| SWARS1 | *Swartzia* | *simplex_var.grandiflora* | Fabaceae: Caesal. | S | Mam |
| SWARS2 | *Swartzia* | *simplex_var. ochnacea* | Fabaceae: Caesal. | S | Bird |
| TAB2AR | *Tabernaemontana* | *arborea* | Apocynaceae | I | Mam |
| TET2PA | *Tetragastris* | *panamensis* | Burseraceae | S | Bird |
| TRI2PA | *Trichilia* | *pallida* | Meliaceae | S | Mam |
| TRI2TU | *Trichilia* | *tuberculata* | Meliaceae | S | Mam |
| UNONPI | *Unonopsis* | *pittieri* | Annonaceae | S | Bird |
| VIROSE | *Virola* | *sebifera* | Myristicaceae | S | Mam |
| XYL1MA | *Xylopia* | *macrantha* | Annonaceae | S | Bird |
| ZANTBE | *Zanthoxylum* | *ekmanii* | Rutaceae | G | Bird |

***Table C2:*** Results of the permutation tests of independence between dispersal agent (bat, bird, and mammal as individual categories of animal dispersers) and spatial patterns at small- (2-10m) and large-scale (60-100m) distance intervals.

|  | **dispersal agent: small scale** | | | | **dispersal agent: large scale** | | | |
| --- | --- | --- | --- | --- | --- | --- | --- | --- |
|  | independence | segregation | partial overlap | mixing | independence | segregation | partial overlap | mixing |
| Bat | -1.14 | -1.04 | -0.39 | 2.12 | -1.18 | -0.70 | -1.12 | 1.99 |
| Bird | 1.61 | -4.14 | -0.33 | 1.13 | -3.03 | -2.74 | 1.46 | 3.66 |
| Exp | -4.36 | -1.48 | 2.75 | 5.51 | 0.11 | -0.60 | -0.95 | 0.52 |
| Mam | 1.47 | 1.28 | -0.88 | -2.44 | 2.41 | 3.48 | -0.42 | -3.44 |
| Wind | -3.93 | 10.32 | -0.41 | -2.99 | 3.76 | -0.73 | -1.16 | -3.21 |
| *maxT* = 10.3226, *p* < 2.2e-16 | | | | | *maxT* = 3.7559, *p* = 0.0247 | | | |

(Note: probability values with *p* < 0.05 reject the null hypothesis of independence and indicate that the four types of association between adults and recruits depend on the dispersal agent. Large positive or negative values in the standardized contingency table highlight deviation from independence in favour of a positive or negative association between spatial patterns and the five dispersal modes bat, bird, explosive, mammal, and wind.)

***Appendix D:*** *Analyses of adult-recruit associations using only recruits of the 2010 census*

**Figure D1:** Spatial pattern analysis of adult-recruit associations and their scale-dependent changes. Unlike in the main analysis, here only the recruits of 2010 and their potential parent trees of step-wise earlier censuses (adults 2005-1982) are analysed in order to assess the effect of the temporal variation in maturation or senescence of the potential parent cohort. Independence made up 70.0%, 66.7%, 70.0%, 73.3%, 66.7%, and 63.3% of the adult-recruit pairs in the 2010, 2005, 2000, 1995, 1990, and 1985 censuses, respectively.

| 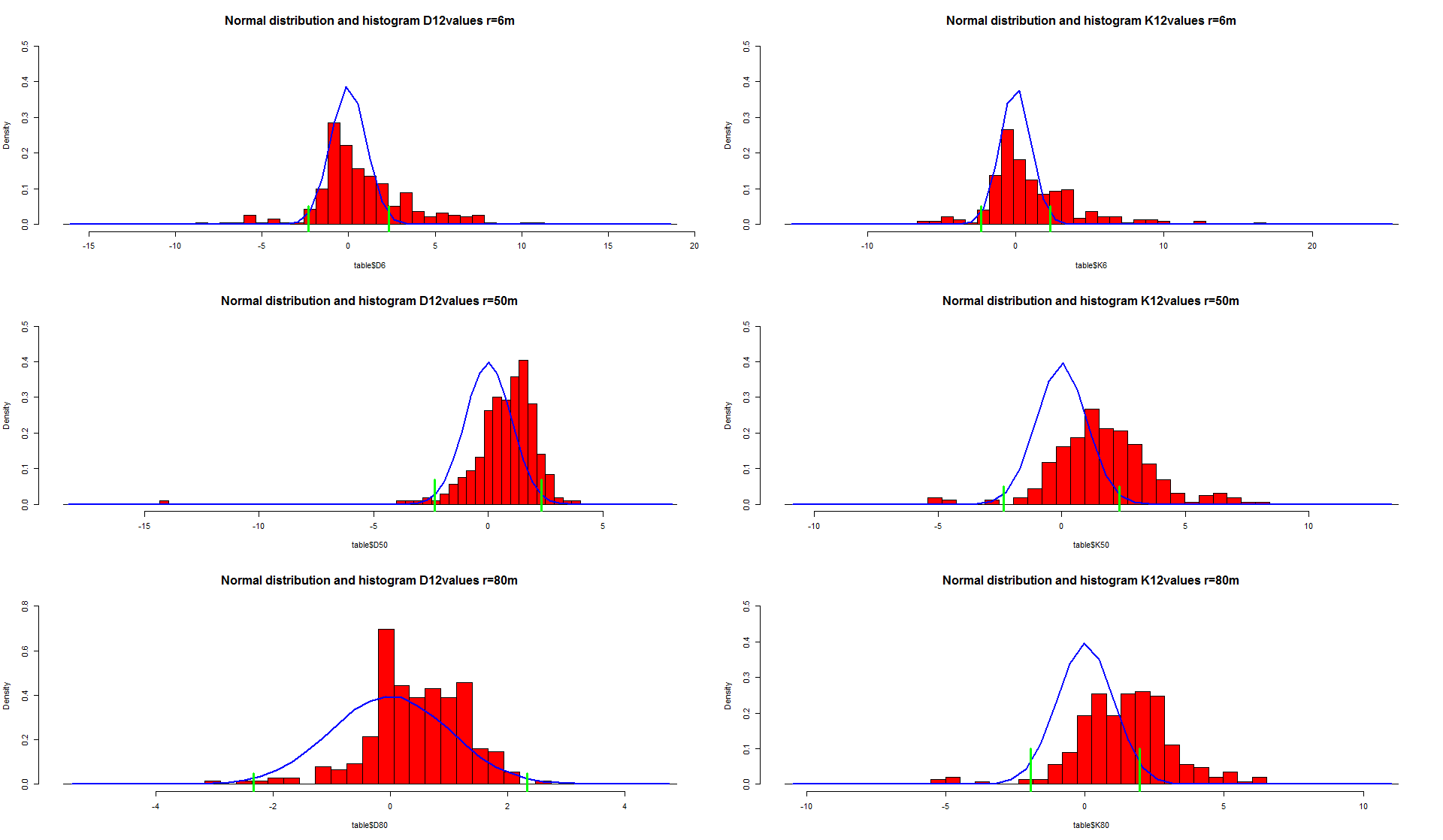 |
| --- |
| **Figure D2**: Distribution of the effects sizes *P*(*r*) and *D*(*r*) for neighbourhood scales of 6, 50 and 80m. To outline the strength of the effects we also show the standard normal distribution (blue). Values between -2.33 and 2.33 (green) correspond to a P-value of 0.025 for two summary statistics individually and encircle cases without significant departures of the null model. |

***Appendix E:*** *Analyses of habitat association*

The dependency of species on the spatial covariates was generally low. The most important covariate was slope, followed by TWI, and elevation. Summarized as averages over all six censuses, the percentages of species where adults or recruits showed a significant association to a topographic covariate yielded 23.9 and 26.6 (slope), 24.4 and 23.4 (topographical wetness index; TWI), 25.2 and 19.3 (elevation), 20.7 and 12.5 (aspect), 14.4 and 11.8 (convexity), and 8.4 and 11.7 (vertical distance to streams; VDS), respectively (figure E1a). Summarized in the same way, 18.8% of all species showed on average no significant association to any of the six topographic covariates.

To get a clearer picture on the overall dependency on covariates, we also analysed the percentages of species whose adults and recruits were both at the same time dependent on the same covariate. Summarized as averages over all six censuses the percentages of species consistently associated with slope were 13.7, with TWI 10.9, elevation 7.6, aspect 6.4, VDS 1.8, and with convexity were 1.6 (figure E1 b). Thus, on average only one fourth of the species of the aforementioned analysis still showed significant habitat association. For those species whose adults and recruits were both dependent on the same covariate, we additionally assessed how many of them were dependent in the same direction and in opposed direction. Adults and recruits of the same species showed in most cases the same type of response to a covariate (e.g. 13.4% for slope or 10.5% for TWI). Only a total of five species showed in some years an opposite response to a covariate: for example, adults of *Alseis blackiana* were negatively associated with slope but recruits positively.

|  |
| --- |
| **Figure E1:** Berman test of association with the spatial covariates elevation (Elv), slope (S), aspect (Asp), convexity (Con), topographical wetness index (TWI), and vertical distance to streams (VDS) showing all those significant cases with tree patterns being an inhomogeneous Poisson point process with their intensity depending on the distance from the covariate. Figure 3(a) shows the percentages of species that have their adults or recruits associated with a covariate while 3(b) presents only those species where both, adults and recruits depend together on the same covariate. |

| 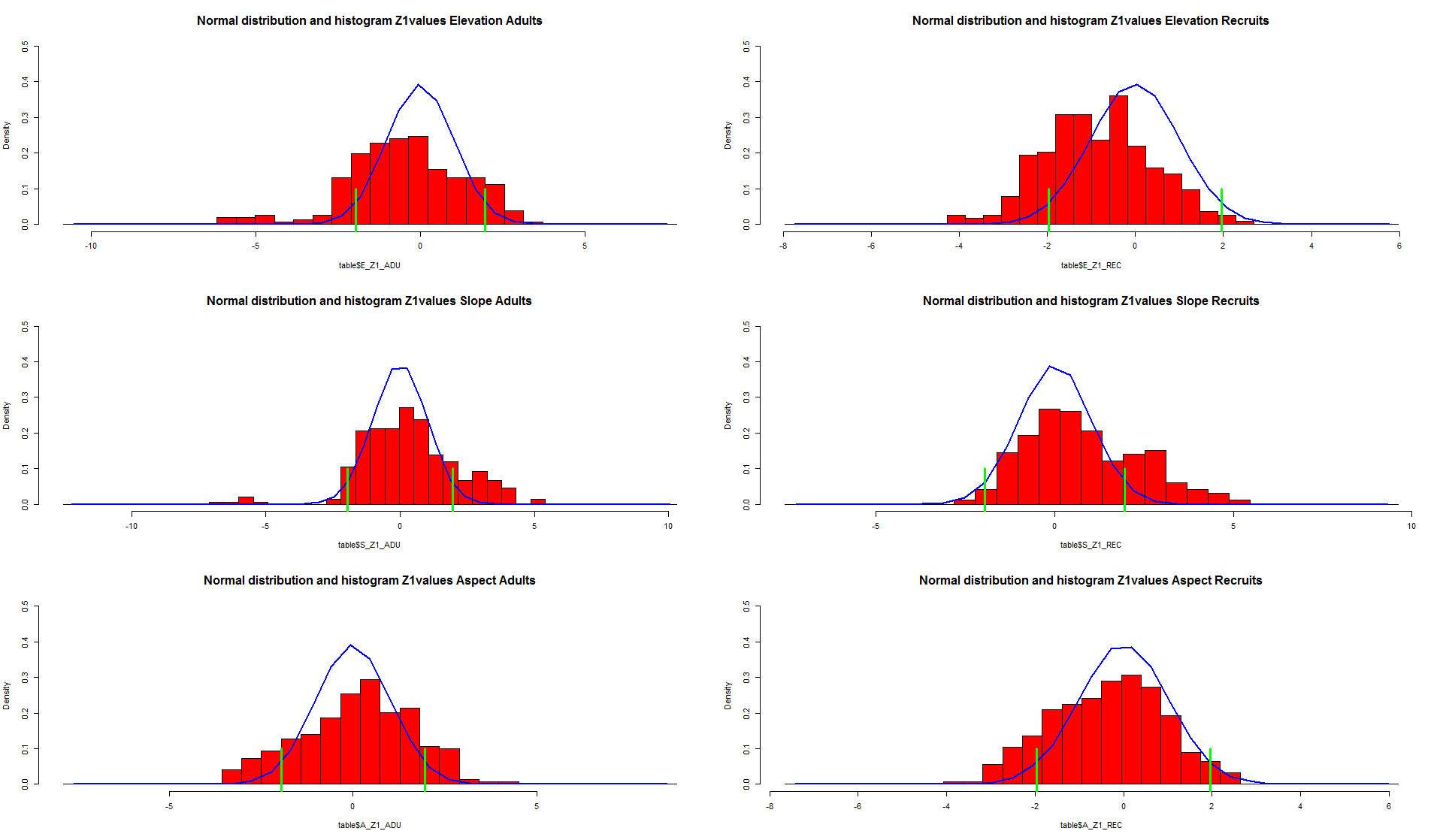 |
| --- |
| **Figure E2**: Distribution of the effects size summary statistic *Z*_1_ of the Berman test for the different species and environmental covariates elevation, slope, and aspect. To outline the strength of the effects we also show the standard normal distribution (blue). Values between -1.96 and 1.96 (green) correspond to a P‑value of 0.05 and encircle cases without significant departures of the null model. |

| 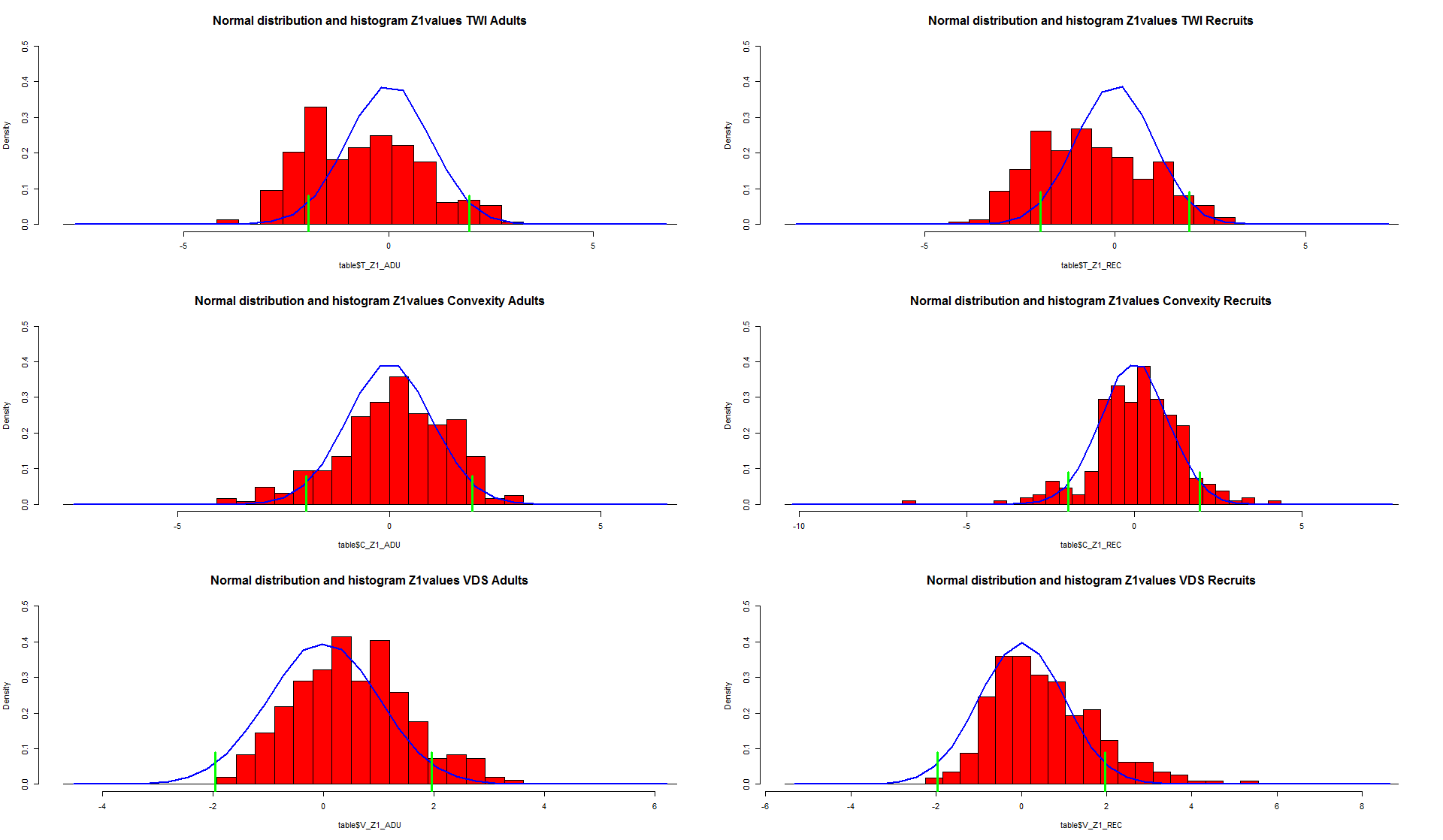 |
| --- |
| **Figure E2**: Distribution of the effects size summary statistic *Z*_1_ of the Berman test for the different species and environmental covariates topographic wetness index (TWI), convexity, and vertical distance to streams (VDS). To outline the strength of the effects we also show the standard normal distribution (blue). Values between -1.96 and 1.96 (green) correspond to a P‑value of 0.05 and encircle cases without significant departures of the null model. |

***Table E.*** Spearman rank correlation coefficients *rho* between the number of stems of recruits (*n*_Recruits_) and adults (*n*_Adults_), respectively, and the ranks of the *p*-values of the Berman tests of species association with the spatial covariates: elevation (Elv), slope (S), aspect (Asp), convexity (Con), topographical wetness index (TWI), and vertical distance to streams (VDS). Negative values of *rho* indicate that there was a negative correlation between the number of stems and the *p*-values of the Berman tests. Besides *rho*, shown are also the *p*-values of the Spearman rank correlation. The Spearman rank correlation coefficients between the ranks of the *p*-values of the Berman tests of species association with the spatial covariates and the number of stems per species were very low. The absolute values of the correlation coefficients were mainly smaller than 0.3, indicating that sample size had a weak effect when testing for habitat association.

| spatial  covariate | *n*_Recruits_ *rho* | *n*_Recruits_  *p*-value | *n*_Adults_  *rho* | *n*_Adults_  *p*-value |
| --- | --- | --- | --- | --- |
| Elv | -0.170 | 0.004 | -0.287 | 0.000 |
| S | -0.169 | 0.005 | -0.268 | 0.000 |
| Asp | -0.264 | 0.000 | -0.377 | 0.000 |
| Con | -0.063 | 0.298 | -0.256 | 0.000 |
| TWI | -0.045 | 0.459 | -0.019 | 0.750 |
| VDS | -0.022 | 0.717 | -0.069 | 0.251 |

***Appendix F:*** *Relationship between adult-recruit associations and sample size*

To find out if the significance of our results was dependent on the number of individuals of the adult-recruit pairs we followed the approach taken in Wiegand *et al.* (2012). We calculated for all adult-recruit pairs the Spearman rank correlation between the rank *u*_0_ of the goodness-of-fit test and the number *n*_Adults_ of individuals of adults and the number *n*_Recruits_ of individuals of recruits at various distances *r*. We conducted the goodness-of-fit test for the same single neighbourhoods *r* = 2m, 6m, 10m (small scale), and *r* = 60m, 80m, 100m (large scale) as used in the permutation tests because these scales represent neighbourhoods where deviations from independence were most pronounced (i.e., mixing and partial overlap peaked, respectively, *cf.* figure 2). We tested the correlation between the ranks of the goodness-of-fit tests of the *M* axis (which is related to the total number of recruit neighbours within distance *r* around the individuals of the adults) at different neighbourhoods *r* and the adult number *n*_Adults_ and recruit number *n*_Recruits_, respectively. This Spearman rank correlation was also repeated for the rank of the overall GoF test applied for the distance interval *r* = 1- 250m (*cf.* analysis 1) to test if the number of individuals of adults or recruits, respectively, did affect the overall deviation from independence (as measured with the ranks for the *M* and *P* axes).

***Table F4.*** Spearman rank correlation coefficients *rho* between the ranks of the goodness-of-fit tests of the *M* axis for the six single small- and large-scale neighbourhoods *r* and the number of stems of recruits (*n*_Recruits_) and adults (*n*_Adults_), respectively. Below are also shown the coefficients *rho* for the overall GoF test (1 - 250m), as measured with the ranks for the *M* and *P* axes. Besides *rho*, shown are also the *p*-values of the Spearman rank correlation. The results show that only at very small neighbourhoods of *r* = 2m, deviations from independence were slightly affected by the number of recruits or adults, respectively.

| ranks of  GoF test | *n*_Recruits_ *rho* | *n*_Recruits_  *p*-value | *n*_Adults_  *rho* | *n*_Adults_  *p*-value |
| --- | --- | --- | --- | --- |
| *M*(*r =* 2m) | 0.371 | 0.000 | 0.346 | 0.000 |
| *M*(*r =* 6m) | 0.039 | 0.521 | 0.034 | 0.571 |
| *M*(*r =* 10m) | -0.010 | 0.869 | 0.003 | 0.959 |
| *M*(*r =* 60m) | -0.091 | 0.130 | 0.099 | 0.099 |
| *M*(*r =* 80m) | -0.082 | 0.173 | 0.095 | 0.112 |
| *M*(*r =* 100m) | -0.121 | 0.044 | 0.068 | 0.257 |
| *M*(*r =* 1 - 250m) | -0.129 | 0.032 | 0.032 | 0.598 |
| *P*(*r =* 1 - 250m) | -0.031 | 0.606 | 0.133 | 0.027 |

**References**

Beckman, N. G., Muller-Landau, H. C. 2007 Differential effects of hunting on pre-dispersal seed predation and primary and secondary seed removal of two neotropical tree species. *Biotropica* **39**, 328–339.

Berman M. 1986 Testing for spatial association between a point process and another stochastic process. *Appl Stat-J Roy St C* **35**(1), 54-62. (doi:Doi 10.2307/2347865).

Comita, L. S., Aguiar, S., Pérez, R., Lao, S., Hubbell. S. P. 2007 Patterns of woody plant species abundance and diversity in the seeding layer of a tropical forest. *J Veg Sci* **18**, 163–174.

Dalling, J., **Muller-Landau**, H. C., Wright, S. J., Hubbell, S. P. 2002 Role of dispersal in the recruitment limitation of neotropical pioneer species. *J Ecol* **90**, 714-727.

Goldsmith, G., Zahwahi, R. 2007 The function of stilt roots in the growth strategy of Socratea exorrhiza (Arecaceae) at two neotropical sites. *Rev. Biol. Trop.* **55**, 787-793.

Harms K.E., Condit R., Hubbell S.P., Foster R.B. 2001 Habitat associations of trees and shrubs in a 50-ha neotropical forest plot. *J Ecol* **89**(6), 947-959. (doi:DOI 10.1046/j.0022-0477.2001.00615.x).

Hothorn T., Hornik K., Van de Wiel M.A., Zeileis A. 2006 A Lego system for conditional inference. *Am Stat* **60**(3), 257-263. (doi:Doi 10.1198/000313006x118430).

Illian J., Penttinen A., Stoyan H., Stoyan D. 2008 *Statistical analysis and modelling of spatial point patterns*. Chichester, England ; Hoboken, NJ, John Wiley; xix, 534 p. p.

John R., Dalling J.W., Harms K.E., Yavitt J.B., Stallard R.F., Mirabello M., Hubbell S.P., Valencia R., Navarrete H., Vallejo M., et al. 2007 Soil nutrients influence spatial distributions of tropical tree species. *P Natl Acad Sci USA* **104**(3), 864-869. (doi:DOI 10.1073/pnas.0604666104).

Kirkpatrick, S., Gelatt Jr., C. D., Vecchi, M.P. 1983 Optimization by simulated annealing. *Science* **220**, 671-680.

Lotwick H.W., Silverman B.W. 1982 Methods for analyzing spatial processes of several types of points. *J Roy Stat Soc B Met* **44**(3), 406-413.

Muller-Landau H.C., Wright S.J., Calderon O., Condit R., Hubbell S.P. 2008 Interspecific variation in primary seed dispersal in a tropical forest. *J Ecol* **96**(4), 653-667. (doi:DOI 10.1111/j.1365-2745.2008.01399.x).

Plotkin, J.B., Potts, M.D., Leslie, N., Manokaran, N., LaFrankie, J. & Ashton, P.S. 2000 Species-area curves, spatial aggregation, and habitat specialization in tropical forests. *J. Theor. Biol*. **207**, 81–99.

(doi:10.1006/jtbi.2000.2158)

Tscheschel A., Stoyan D. 2006 Statistical reconstruction of random point patterns. *Comput Stat Data An* **51**(2), 859-871. (doi:DOI 10.1016/j.csda.2005.09.007).

Wiegand T., Moloney K.A. 2004 Rings, circles, and null-models for point pattern analysis in ecology. *Oikos* **104**(2), 209-229. (doi:DOI 10.1111/j.0030-1299.2004.12497.x)

Wiegand, T, Gunatilleke, C.V.S., Gunatilleke, I.A.U.N., Okuda, T. 2007 Analyzing the spatial structure of a Sri Lankan  tree species with multiple scales of clustering. *Ecology* **88**, 3088–3102.

Wiegand T., Martinez I., Huth A. 2009 Recruitment in tropical tree species: revealing complex spatial patterns. *Am Nat* **174**(4), E106-E140. (doi:Doi 10.1086/605368).

Wiegand T., Huth A., Getzin S., Wang X.G., Hao Z.Q., Gunatilleke C.V.S., Gunatilleke I.A.U.N. 2012 Testing the independent species' arrangement assertion made by theories of stochastic geometry of biodiversity. *P Roy Soc B-Biol Sci* **279**(1741), 3312-3320. (doi:DOI 10.1098/rspb.2012.0376).

Wiegand T., He F.L., Hubbell S.P. 2013 A systematic comparison of summary characteristics for quantifying point patterns in ecology. *Ecography* **36**(1), 92-103. (doi:DOI 10.1111/j.1600-0587.2012.07361.x).

Wright, S.J. 2011 Tropical plant reproduction biology. [WWW document]. URL http://www.stri.si.edu/sites/esp/tesp/plant_species_a.htm
